# Antimicrobial Peptides—Mechanisms of Action, Antimicrobial Effects and Clinical Applications

**DOI:** 10.3390/antibiotics11101417

**Published:** 2022-10-16

**Authors:** Jasminka Talapko, Tomislav Meštrović, Martina Juzbašić, Matej Tomas, Suzana Erić, Lorena Horvat Aleksijević, Sanja Bekić, Dragan Schwarz, Suzana Matić, Marijana Neuberg, Ivana Škrlec

**Affiliations:** 1 Faculty of Dental Medicine and Health, Josip Juraj Strossmayer University of Osijek, 31000 Osijek, Croatia; 2 University Centre Varaždin, University North, 42000 Varaždin, Croatia; 3 Institute for Health Metrics and Evaluation, University of Washington, 3980 15th Ave. NE, Seattle, WA 98195, USA; 4 Faculty of Medicine, Josip Juraj Strossmayer University of Osijek, Josipa Huttlera 4, 31000 Osijek, Croatia; 5 Family Medicine Practice, 31000 Osijek, Croatia

**Keywords:** antimicrobial peptides, antimicrobial resistance, antimicrobial effects, antibacterial activity, antiviral activity, antifungal activity, microorganisms, clinical trials, infections, treatment

## Abstract

The growing emergence of antimicrobial resistance represents a global problem that not only influences healthcare systems but also has grave implications for political and economic processes. As the discovery of novel antimicrobial agents is lagging, one of the solutions is innovative therapeutic options that would expand our armamentarium against this hazard. Compounds of interest in many such studies are antimicrobial peptides (AMPs), which actually represent the host’s first line of defense against pathogens and are involved in innate immunity. They have a broad range of antimicrobial activity against Gram-negative and Gram-positive bacteria, fungi, and viruses, with specific mechanisms of action utilized by different AMPs. Coupled with a lower propensity for resistance development, it is becoming clear that AMPs can be seen as emerging and very promising candidates for more pervasive usage in the treatment of infectious diseases. However, their use in quotidian clinical practice is not without challenges. In this review, we aimed to summarize state-of-the-art evidence on the structure and mechanisms of action of AMPs, as well as to provide detailed information on their antimicrobial activity. We also aimed to present contemporary evidence of clinical trials and application of AMPs and highlight their use beyond infectious diseases and potential challenges that may arise with their increasing availability.

## 1. Introduction

Antimicrobial peptides (AMPs) have played a key role in numerous scientific studies intending to find new antimicrobial agents and active substances [1]. The need for new antimicrobial drugs is increasingly recognized, driven by the growing global burden of antimicrobial resistance and a significant increase in infections that cannot be treated with existing antibiotics [2,3,4]. A particular problem is the ESCAPE group of pathogens, which includes *Escherichia coli*, *Staphylococcus aureus*, *Klebsiella pneumoniae*, *Streptococcus pneumoniae*, *Acinetobacter baumannii*, and *Pseudomonas aeruginosa*, which, according to data from 2019, are responsible for more than 250,000 antimicrobial-related deaths resistance [5]. Antimicrobial resistance is associated with high mortality rates. In addition, it significantly impacts the effectiveness of antimicrobial agents as it reduces the efficacy of treatment and prolongs the time of infection in patients [5]. Additionally, another group of pathogens associated with antimicrobial resistance is responsible for another 100,000 to 250,000 deaths, consisting of *M. tuberculosis*, *Enterococcus faecium*, *Enterobacters* spp., *Streptococcus agalactiae*, *S. typhi*, and *Enterococcus faecalis*. All this influenced the increase in treatment costs due to the use of more expensive therapies [5]. It is important to note that a significantly higher degree of resistance is present in Gram-negative bacteria. Accordingly, treating infections caused by this group of pathogens is more complicated. Namely, many first-line antibiotics, such as vancomycin, rifampicin, and others that are successful in treating infections caused by Gram-positive pathogens due to their ineffectiveness against Gram-negative pathogens, are not used in the treatment of infections caused by this group of pathogens. The problem is the permeability of the lipopolysaccharide (LPS) of the outer membrane and porins because they largely limit the entry of antibiotics into the cell [6]. Gram-negative opportunistic pathogens could be resistant to commercial antibiotics due to the LPS of the outer membrane [7]. Cationic AMPs provide new possibilities in treating infections caused by Gram-negative pathogens because they can often permeabilize the anionic LPS of the outer membrane, which leads to cell lysis and opens the way to action on Gram-negative bacteria. Likewise, AMPs can act as enhancers of the antimicrobial activity of conventional antibiotics on Gram-negative pathogens [8]. However, some Gram-negative bacteria have an innate resistance to cationic AMPs [9] associated with changes in the LPS of the outer membrane [7,9]. AMPs are known to be widespread [10]; they are involved in innate immunity and represent the first line of defense against pathogens in the host [11]. They show a wide range of antimicrobial activity against Gram-negative and Gram-positive bacteria, fungi (particularly yeasts), and viruses [12,13]. Significantly, they have a low tendency to induce drug resistance, thus providing us with the opportunity to use them as a specific class of antibiotics [14,15]. However, we have to be cognizant that the organism’s protection from pathogens depends on how they distribute in tissues and whether external administration is free from toxicity issues [16].

## 2. Antimicrobial Peptides

Antimicrobial peptides are composed of a different number of amino acids. This number is usually from 10 to 60 amino acids [13]. Amphipathic properties have a vital role in the activity of AMPs [17]. Thanks to this property, AMPs are easily integrated into the cell membrane or pass through into the cytosol [18]. The mechanisms of action on microorganisms depend on different physicochemical properties: charge, structure, sequence length, peptide concentration, hydrophobicity, and membrane composition [19]. Ribosomally synthesized AMPs that contain only natural amino acids are divided into linear, α-helical peptides (such as cecropins, magainins, and melittins), peptides labeled with enrichment of one or two amino acids (PR 39 rich in proline-arginine, indolidin), and peptides containing disulfide bonds (e.g., defensins, protegrin) [20]. Additionally, peptides with strong antimicrobial activity synthesized extraribosomally have significant posttranslational modifications, such as lipopeptides (polymyxin, dermaseptin) and lantibiotics containing non-native amino acids [21].

### Mechanism of Action of Antimicrobial Peptides

**Figure 1:**
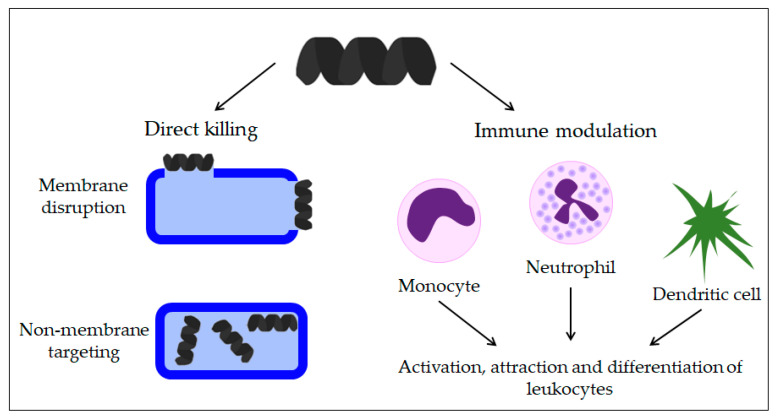
Mechanisms of AMPs action.

Research suggests that AMPs have a completely different mechanism of action on microorganisms than antibiotics currently used to treat infections [1,22,23] (Figure 1). 

**Figure 2:**
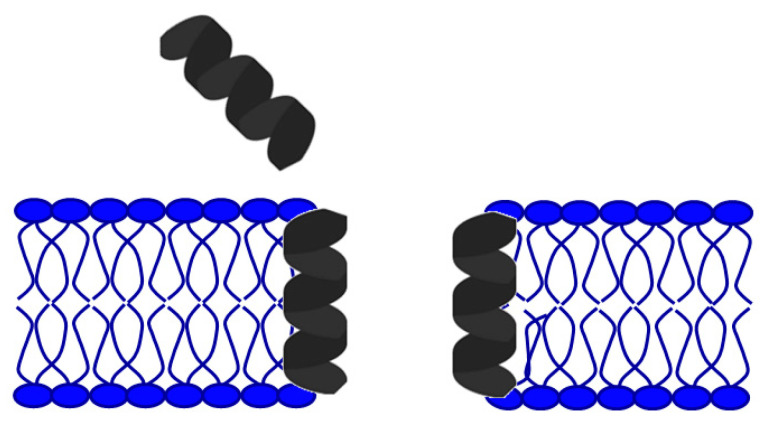
Barrel–stave pore model.

Understanding the interaction of peptides with microorganism membranes is a significant factor in improving the design and development of AMPs [24]. The interaction of these peptides with biological membranes and their structure also depends on the lipids contained in the cell membrane itself [25]. Individual AMPs interact with the bacterial cell membrane and thus interfere with the construction of the inner or outer bacterial membrane, resulting in cell death [26]. Impaired membrane integrity occurs due to the interaction of AMP with a negatively charged cell membrane, inhibition of protein, DNA, and RNA synthesis, or interaction with specific intracellular targets [22,24,27]. The critical interaction points between the peptide and the bacterial membrane are electrostatic forces between the cationic AMPs and the negatively charged bacterial surface [28,29,30]. The cytoplasmic membranes of Gram-positive and Gram-negative bacteria are rich in phospholipids, phosphatidylglycerol, and cardiolipin, which have negatively charged major groups which strongly attract positively charged AMPs [1,31]. The cationic amphipathic α-helix is one of the most common types of AMP [25]. For amphipathic α-helix peptides, several models explain how they work. The barrel pore model (Figure 2) in which the amphipathic α-helix creates vertical pores across the membrane and peptides accumulate in barrel-shaped aggregates showing water-permeable and transmembrane-oriented pores [32,33].

**Figure 3:**
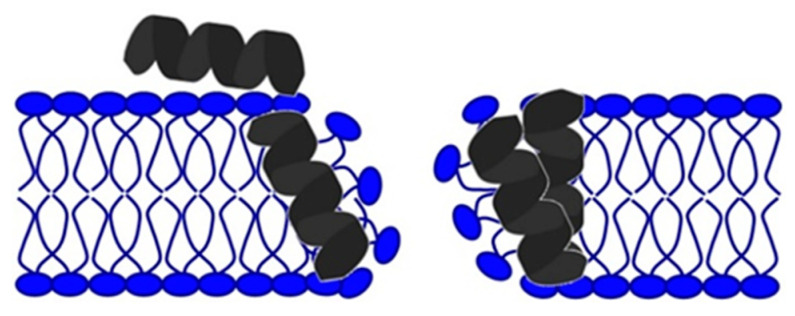
Toroidal pore model.

The formation of a transmembrane water-permeable pore results from the association of several AMP molecules with lipid heads in a toroidal pore model (Figure 3) [33].

**Figure 4:**
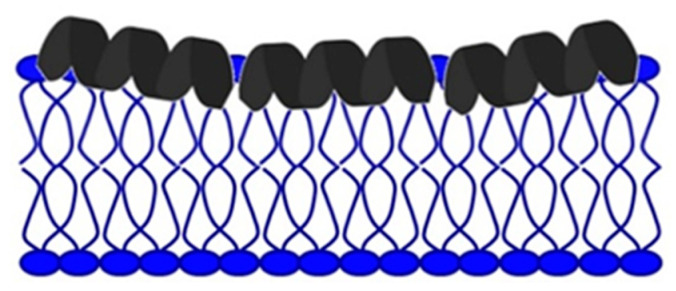
Carpet model.

The carpet model (Figure 4) provides an alternative view where AMPs come into contact with the phospholipid head and spread across the membrane surface, covering the carpet-like membrane. When the peptide concentration reaches a critical value in this model, the membrane collapses, creating defects, and dissolves into micelles [33].

## 3. Antimicrobial Effects of AMPs

### 3.1. Antibacterial Activity of AMPs

**Figure 5:**
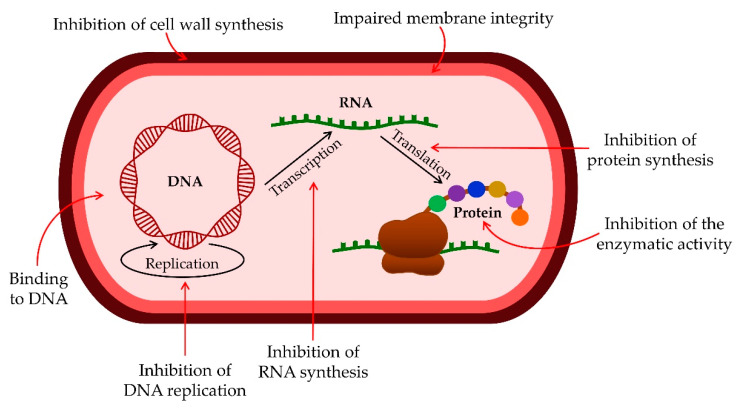
Antibacterial activity mechanisms of AMPs.

As the primary goal in researching AMPs was the fight against (increasingly resistant) bacteria, some scientists emphasize this need and call them antibacterial peptides (ABP) [34,35]. They can destroy bacteria by breaking the cell wall and membrane of the bacteria, intracellular action, through a combination of dual destruction mechanisms, and by acting on the bacterial biofilm [36,37]. By binding to lipid II, which is part of the peptidoglycan molecule and an essential factor in cell wall synthesis, ABPs prevent cell wall formation. In addition to controlling its assembly, it can destroy the already-formed cell wall [37,38,39,40]. Acting on the membrane is possible in several ways. The barrel-shaped and toroidal pore models can be defined as transmembrane pore models. Vertical insertion of the peptide into the membrane while promoting peptide–peptide lateral interaction is typical of the barrel-shaped pore model [37,41,42]. In the toroidal pore model, peptides are also vertically embedded in the membrane. However, here the pores are created by both peptide and lipid groups. The most crucial difference between these two models is that the barrel model’s hydrophobic and hydrophilic bilayers remained intact [37,42,43]. The model that does not create pores through the membrane and in which specific peptide–peptide interactions are not necessary belongs to the so-called carpet model. It is characterized by adherence of ABP along the bacterial membrane and its adsorption until a particular concentration is reached. After crossing the tolerance threshold, a detergent-like model appears, and a loss of integrity and opening of the bacterial membrane happens [13,37,43,44,45]. Many peptides work by destroying intracellular functions, and in that way, they kill bacteria. Some important ones inhibit DNA, RNA, and protein synthesis [42,46,47,48]. The development of research and the discovery of peptides with antimicrobial effects show that specific peptides do not have only one impact model (Figure 5). On the contrary, most of them share a combination of mechanisms [37,42,43,49,50].

AMPs can act on biofilm at all stages of its development. They can inhibit biofilm formation by disrupting the signaling pathway of bacteria cells. By encouraging bacteria to produce guanosine tetraphosphate (ppGpp) and pentaphosphate (pppGpp) with limiting nutrients in the biofilm, the synthesis of nucleic acids is inhibited. Another effect is a reduction in the expression of binding protein transport genes necessary for forming a bacterial biofilm. Additionally, ABP can destroy the already-formed biofilm by acting on the membrane potential of bacteria [37,43,51,52].

### 3.2. Antiviral Activity of AMPs

Due to the increasing resistance of viruses and the limited effect of common drugs, antiviral peptides play an essential role as therapeutic agents [43]. AMPs with antiviral activity are referred to as antiviral peptides (AVPs) [53,54]. There are several different mechanisms by which antiviral peptides block viruses at various stages of their cycle [34]. Some AVPs can destabilize the viral membrane or prevent infection by neutralizing the virus by integrating it into the viral envelope and cell membrane [2,55]. Similarly, they can bind to viral glycoproteins, after which the viruses can no longer bind to the host cell surface [2,56], while some bind to host cell receptors, preventing the virus from binding to its target receptor and thus inactivating it (e.g., heparan for herpes simplex virus). Some block viral attachment and prevent membrane fusion in the virus cell (e.g., influenza virus) [55,57]. An essential mechanism of action of AVP on the influenza virus is the regulation of the human immune system with the increased expression of cytokines and chemokines on the antigen complex, activation of cells of the immune system, and inactivation of viral pathogens (e.g., H1N1 virus) [58,59,60]. In addition, AVP blocks its activity by acting on the replication cycle [61]. AVP shows similar effects to those described for influenza virus in inhibiting human immunodeficiency virus 1 (HIV-1), herpes simplex virus 1 (HSV-1), herpes simplex virus 2 (HSV-2), hepatitis B virus (HBV), and hepatitis C virus (HCV) infections [61]. The target site of AVPs can be DNA and RNA [62,63], and the goal of their action is to destroy the viral envelope, leading to its instability, as in the Junin virus (JV), HIV-1, and HSV-2 [64]. Another way to inhibit viruses is to modify or interfere with cell signaling pathways [65], such as GF-17 (17-mer-derived peptide from human cathelicidin LL-37) and BMAP-18 (bovine myeloid antimicrobial peptide-18), which can inactivate the Zika virus (ZIKV) by interfering with interferon type 1 signaling [54]. Recently, there has been an increasing number of studies on the antiviral effect of AVPs on coronaviruses (such as SARS-CoV). In short, the current dogma of this type of research endeavors is based on AVP’s inhibitory effect on the viral cell membrane and its inability to enter the host cell [66,67,68,69]. 

### 3.3. Antifungal Activity of AMPs

The excessive and improper use of systemic antibiotics, immunosuppressive therapy, chemotherapy, and radiotherapy have increased fungal infections in the general population [70,71,72]. The limited choice of antifungal therapy, which includes only four chemical types of systemic antifungals—polyenes, azoles, echinocandins, and flucytosines [71,73]—as well as the increase in the resistance of specific strains to the mentioned antifungal drugs, leads to increased morbidity and mortality, but also a dire need for alternative solutions in antifungal treatment [70,71,74].

Antifungal peptides (AFPs) could be a promising therapy for fungal infections [71,75]. Most AFPs achieve their activity through membrane-associated mechanisms and specific targets. Differences in fungal membranes are sphingolipid composition, PI content, and ergosterol as the main membrane sterol. Specific targets such as glucosylceramides, mannosyldiinositol phosphorylceramide, or a fungal protein target enable high selectivity and avoid resistance to therapy [76].

AFPs can be classified in accordance with a number of criteria, such as structure, mode of action, or origin [77]. However, the most accepted classification is according to the origin, which divides peptides into natural, semisynthetic, and synthetic ones [78].

Natural AFPs are produced by different species like Archaea, Bacteria, and Eukaryotes [77]. Natural AFPs have an α-helix structure, β-hairpin or sheet, or combination of α-helix and β-sheet, and depending on the amino acid in the most significant composition. They are classified as glycine-rich, arginine-rich, proline-rich, histidine-rich, and tryptophan-rich peptides [79].

Semisynthetic and synthetic peptides are designed to improve pharmacological properties and reduce immunogenicity and side effects caused by natural peptides [77]. Biophysical characteristics such as net charge, stereospecificity, hydrophobicity, secondary structure, peptide length, and amphipathicity determine the antifungal activity of peptides [80]. For example, an increase in the positive net charge can cause a stronger action on the membrane. Likewise, increased hydrophobicity and amphipathicity lead to increased antifungal activity [77].

The most abundant peptides harmful to the fungal biofilm are peptides of mammalian origin, defensins, cathelicidins, and histatins [71]. Defensins are isolated from not only mammals but also plants [81]. They are structurally organized as α-helix and triple-stranded antiparallel β-sheet, which are connected by disulfide bonds that ensure the stability of the structure, even in extreme conditions [82]. Defensins from vertebrate animals are cationic and amphipathic peptides and can be divided into two subfamilies, α-defensins and β-defensins [83]. Human α-defensin 6 (HD6) prevents the adhesion of *Candida albicans* (*C. albicans*) to human intestinal epithelial cells and, thus, biofilm formation [84]. β-defensin-1 displays inhibitory activity against germinating conidia of *Aspergillus fumigatus* [85]. Synthetic defensin-like peptides like α-defensin-3, β-defensin-1, β-defensin-3, and PG-1 express antifungal activity against *Cryptococcus neoformans* biofilms, including both planktonic cells and mature biofilm [86]. Cathelicidins are cationic peptides isolated from different species of mammals, consisting of 12–80 amino acids [71,87]. The human antimicrobial peptide LL-37 was proven to inhibit *C. albicans* cell adhesion on polystyrene and silicon surfaces, and BMAP-28, a bovine antimicrobial peptide, was able to reduce the number of *C. albicans* adherent cells on silicone surfaces and inhibit its mature biofilm [88,89,90]. Histatins are human salivary peptides, first isolated from human parotid saliva, with polar and hydrophilic properties and α-helix structural conformation in organic solutions [91]. Histatin-5 (Hst-5) was proven to inhibit biofilm formation of *C. albicans* on acrylic dentures in vitro [92] and, in another study, inhibit biofilm formation and planktonic cells of *C. albicans* and *Candida glabrata* on methyl methacrylate disks [93].

### 3.4. Immunomodulatory Activity of AMPs

**Figure 6:**
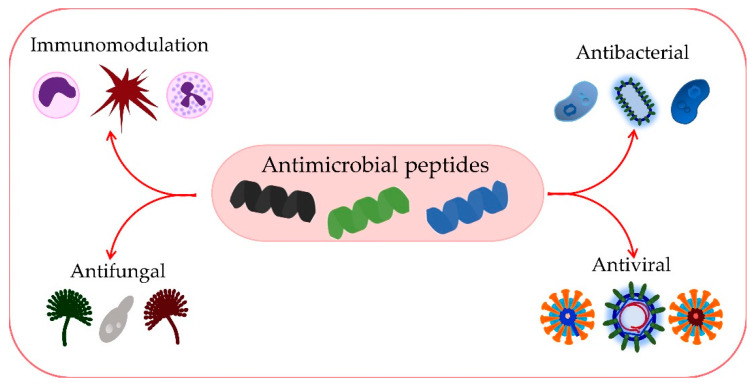
Numerous functions of AMPs.

AMPs play a significant role in immunomodulation and inflammation control [94,95] (Figure 6). The mechanisms of action of AMPs in immune modulation involve various immune responses [96]. The three main families of AMPs in humans are defensins, histatins, and cathelicidins. Based on the arrangement of disulfide bonds, defensins are divided into α-defensins and β-defensins, and they are produced by lymphocytes, neutrophils, and epithelial cells of mucous membranes and skin [24].

#### 3.4.1. Defenses

Since human α-defensins 1-4, most commonly expressed by neutrophils, are called neutrophil peptides 1-4 (HNP) [97], together with lysozyme, proteases, and other proteins, they participate in the destruction of bacterial pathogens. This gives them exceptional importance in the immune system [98]. Human α-defensins 5 and 6 are produced and secreted mainly by Paneth cells located at the base of Lieberkühn’s crypts in the small intestine and by epithelial cells of the male and female genital organs [99]. In addition, some human AMPs (β-defensins, LL-37) can attract immune cells such as white blood cells, dendritic cells, and mast cells [100]. Beta defensins originate from the epithelial cells of the skin and mucosa of mammals [101]. Thus far, six types of human β-defensins have been isolated and designated as hBD 1-6 [102].

Regarding antimicrobial activity and expression level, hBD-1, hBD-2, and hBD-3 defensins were characterized [101]. Epithelial cells constitutively express hBD-1, hBD-2, and hBD-3, but after stimulation by microorganisms and pro-inflammatory cytokines [103]. The role of β-defensin in connecting adaptive and innate immunity is significant [104]. More specifically, hBD-3 has the ability to rapidly enter TLR4-stimulated macrophages and dampen the expression of pro-inflammatory genes [105]. The role of β-defensin peptides is multifunctional, so in addition to the defensive, antimicrobial function, they also have a clearly expressed immunomodulatory function [106]. Since they are ubiquitous across mucosal surfaces, they are considered essential factors in homeostasis and health [107].

The administration of human peptides has been shown to have multiple protective effects in an in vivo model of infection [19]. Furthermore, some AMPs can act as regulatory molecules, as evidenced by in vitro research showing that defensins can attract phagocytes and lymphocytes to the site of infection, induce fibroblast proliferation, and modify ion flow in epithelial cells [108]. In addition, AMPs can induce bacterial lysis, promote macrophage phagocytosis, prevent the spread of infection, induce mitosis of fibroblasts and epithelial cells, and promote fibroblast growth to improve wound healing [1]. Finally, based on the ability of AMPs to stimulate complement activation and the production of cytokines and antibodies, the influence of AMPs on humoral immunity is currently being further evaluated [98].

#### 3.4.2. Histatins

AMPs, histatins, contain large amounts of histidine amino acids [17]. They were isolated from human parotid salivary glands [109] and are characterized by antifungal and antimicrobial properties [110]. Human saliva contains Histatin-1, and Histatin-3, which are derived from the available genes HTN1 and HTN3. They differ in the number of amino acids and molecular weight [111]. Histatin-5 is derived from histatin-3 and contains an N-terminal for which it is highly reactive and has an affinity for binding with metals [112]. They are characterized by antifungal and antimicrobial properties, while the effects of histatin on immune system cells are unknown [113].

#### 3.4.3. Cathelicidins

Cathelicidins are constitutively expressed at low levels in epithelial cells, skin, and mucosal surfaces and are released to a considerable extent in response to infections, especially by granulocytes and mononuclear phagocytes [114]. Cathelicidins in in vitro conditions have an apparent antimicrobial effect on parasites, fungi, bacteria, and enveloped viruses [115]. Cationic cathelicidins have different mechanisms of action on the cell, damaging the integrity of the negatively charged membrane, which results in cell death [116]. They can act on intracellular processes by promoting protein breakdown, weakening enzymes’ role, and affecting RNA and DNA synthesis [117]. Numerous studies have observed that in the presence of glycosaminoglycans, salt, bacterial DNA, and mucin, i.e., under physiological conditions, the action of LL-37 is inhibited even at high peptide concentrations [88,118]. Based on this, we can conclude that, in vivo conditions, most cathelicidins probably do not have a direct bactericidal effect. However, they still have great importance for preventing microbial infections due to their immunomodulatory effect [98,100]. The ability of cathelicidins to attract many adaptive and innate immune cells to inflammatory sites by modulating the expression of chemokines and chemokine receptors should undoubtedly be highlighted [119]. Thus, cathelicidins can act on various immune cells as direct chemoattractants. This effect is enhanced by their indirect impact on inducing the upregulation of chemokines and chemokine receptors on leukocytes [114,119,120].

## 4. Clinical Application of AMPs

### 4.1. The Use of AMPs against Infectious Agents: A Current State of Evidence

Considering their mechanism of action, AMPs can successfully surpass many drawbacks linked to the use of conventional antimicrobials, such as increased rate of multidrug resistance (which is becoming a public health hazard in recent years), as well as certain issues with potential systemic toxicity and their overall activity [121]. The broad spectrum of action and swift antimicrobial effects, with a lower propensity for resistance development, is what makes AMPs emerging and very promising candidates for more pervasive use in the treatment of infectious diseases [22]. Having more than one specific mechanism of antimicrobial activity of certain AMPs (such as thanatin and a synthetic lipopeptide F365) is an advantage in comparison to several cationic AMPs, which opens the door for achieving a true clinical potential against infections caused by multidrug-resistant pathogens [122,123,124]. This is supported by recent studies that concentrated on in vivo characterization and structure–activity relationships of such compounds [122].

**Figure 7:**
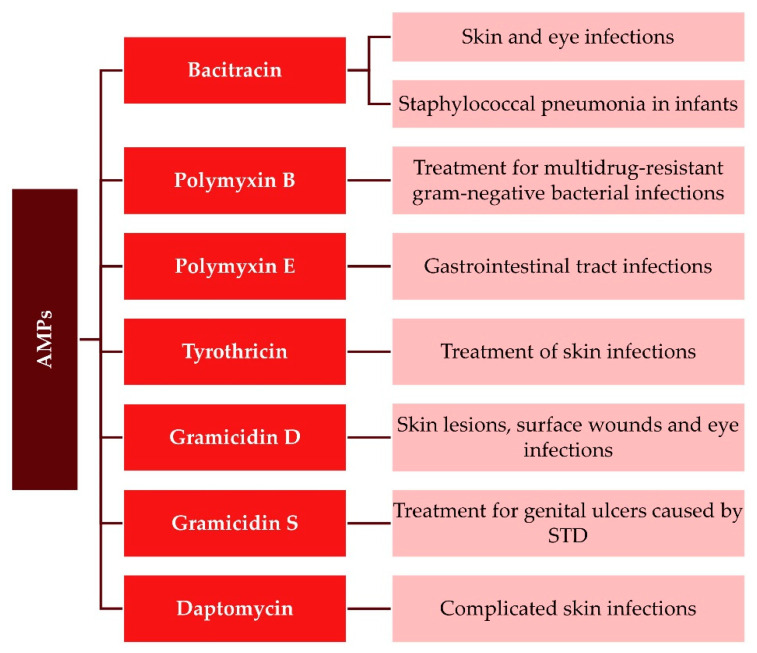
Approved AMPs for use in clinical settings. STD—sexually transmitted diseases.

However, thus far, only a handful of AMPs (from more than three thousand discovered) have been approved for frank clinical usage by the Food and Drug Administration (FDA), and the best-studied ones are gramicidins, polymyxins, and nisins [125,126] (Figure 7). Gramicidins were in restricted use for applications such as infections of surface wounds and the upper respiratory system [127]; conversely, polymyxins can be used not only for treating ocular infections but also for gastrointestinal infections and systemic infections caused by antibiotic-resistant Gram-negative bacterial agents [128]. Another effective cyclic AMP that has its role in treating complicated skin and skin-structure infections as a result of *Staphylococcus aureus* infection is daptomycin, which is often used in combination therapy to improve treatment success rates [129,130]. Dental care, stomach ulcer therapy, and the treatment of colonic infections are known applications of nisins in humans [130,131,132].

Clinical trials appraising the use of linear AMPs (such as pexiganan, omiganan, and DPK060) against different bacterial and fungal infections are currently underway. As an analog of peptide magainin (extracted from the skin of *Xenopus laevis*) with 22 amino-acid residues, pexiganan demonstrates robust antimicrobial activity against bacterial and fungal pathogens, including multiple-resistant ones [125]. Its use as a topical agent in the treatment of foot ulcers that develop in patients with diabetes has been assessed in two phase III clinical trials (CT identifier: NCT01590758, NCT01594762) and also in comparison with the oral formulation (NCT00563433, NCT00563394)—but its spectrum of usage also entails decubital ulcers, as well as infected burns and surgical wounds [133]. Omiganan is a topical cationic 12 amino acid peptide assessed for the treatment of catheter infections, genital warts, rosacea, acne vulgaris, and atopic dermatitis (NCT00321153, NCT03091426, NCT01784133) [125,133]. Kininogen-derived compound DPK060 shows strong and diverse antibacterial activity. Its efficacy as a local emollient in treating acute external otitis and atopic dermatitis has been investigated in phase II clinical trials (NCT01447017, NCT01522391) [134].

A cationic fraction of human lactoferricin, known as hLF1-11, is proposed for intravenous usage in immunocompromised recipients of stem cell transplants for treating both bacterial and fungal infections (NCT00509938) [126,135]. Many other AMPs are in development specifically for fungal diseases; one notable example is a cationic peptide novexatin (generated from defensins) for treating fungal toe infections, while a dimeric peptide CZEN-002 derived from melanocyte-stimulating hormone is another addition to the treatment armamentarium against vaginal candidiasis, an important clinical entity within the vaginitis syndrome [126]. Under clinical trials, there is also POL7080 (Protegrin I) in intravenous infusion for lower respiratory tract infections (NCT02096328) and SPAC-113 (human histatin-3) as a mouth rinse for oral candidiasis in patients with human immunodeficiency virus (HIV) [126,136,137].

### 4.2. Moving beyond Antimicrobial Usage

Several AMPs that are already in advanced stages of clinical development are characterized by mechanisms of action that are not strictly related to their antimicrobial effect (e.g., re-epithelialization and angiogenesis). A salient and well-known example is PXL01, a lactoferrin derivative used to prevent post-surgical adhesions in patients undergoing surgery for flexor tendon repairment, confirmed in phase II trials (NCT01022242) [138]. Such treatment with PXL01, reinforced by important in vitro studies, demonstrated that not only there is no proof of cytotoxicity, but its retained antimicrobial action is also superior to that of lactoferrin [125,138].

Another example is the aforementioned LL-37, with previous preclinical evidence of its involvement in wound healing, although the results were not entirely consistent [125]. An earlier randomized, placebo-controlled trial demonstrated that the topical application of LL-37 can enhance the healing rate of chronic venous leg ulcers [139]. However, that was not corroborated in a newer trial, and it was still concluded that this human synthetic peptide could offer a certain treatment benefit in individuals with large ulcers [140]. Although the exact mechanism of wound repair is still elusive, there is a supposed effect of LL-37 in modulating the inflammatory response and driving angiogenesis together with re-epithelialization [125,141]. Such a wide array of modulatory cell activities also led to the repurposing of LL-37 for cancer treatment [142]. Moreover, its role in oral homeostasis is also known, and it is not related merely to microbiota stabilization [143].

Other clinical studies have evaluated the use of hybrid peptide C16G2 in dental caries in the form of mouth rinse or gel (NCT031962, NCT02044081) [144], porcine protegrin-1 known as iseganan in oral solution for complications in the mouth linked to radiation treatment of head/neck malignancies (NCT000223) [145], and indolicidin in intravenous infusion for oral mucositis that is seen after chemotherapy for squamous cell carcinoma (NCT020130) [146]. In addition, recent studies have shown that regulating the expression of AMPs in gynecological cancers may influence the sensitivity of malignant cells to chemotherapy [147].

In addition to those AMPs administered directly, there are many efforts to boost endogenous AMP synthesis. For example, vitamin D3 has been associated with the upregulation of many AMPs, such as beta-defensins, cathelicidins, and neutrophil gelatinase-associated lipocalin from epithelial cells in the respiratory tract, as well as from macrophages and neutrophils [125,148,149,150]. Furthermore, the increase in plasma levels of free 25-hydroxyvitamin D resulted in increased expression of circulating human cationic antimicrobial protein (hCAP18) mRNA in both healthy and gravely ill ventilator-dependent adult individuals [151,152], with implications for the overall immune response.

### 4.3. Challenges Linked with Clinical Applications—From Screening to Delivery

When designing or striving to optimize AMPs for therapeutic applications against an array of infectious agents, the initial step is to appropriately screen recognized clusters or anticipated peptide sequences for their expected antimicrobial properties. After in silico process (often with the use of cost-effective machine learning) [153], screening is most commonly pursued with the use of standardized and validated assays that aim to measure minimal inhibitory or minimal microbicidal concentration [36]. Nonetheless, differences in environmental conditions represent an important obstacle for corroborating such in vitro findings with in vivo results [22]. Consequently, this can be identified as a crucial hurdle in the more pervasive usage of AMPs for treatment purposes.

As low oral bioavailability and low metabolic stability are inherent traits of oral AMP formulations, this route of administration is not favored, which leaves topical formulations as a preferred option [154]. Likewise, the use of intravenous formulation is hampered by the proteolytic cleavage that takes place in the blood and liver, resulting in a short half-life of such compounds [22]. This means enhancing the stability of AMPs for their broad usage is currently one of the priorities in the pharmaceutical industry, which is already pursued with the use of nanoparticles [155].

Furthermore, as a minimum inhibitory concentration of AMPs (which is arguably the best proxy to evaluate its effectiveness) is habitually lower when compared with conventional antibiotics [156,157], some authors suggest that the optimal AMP molecule should have effects that surpass just antimicrobial activity [158]. One of the solutions would be a multifunctional compound that combines direct activity against pathogenic agents with certain indirect effects, such as immunomodulation. That way, the antimicrobial effect can be enhanced via effective suppression of pro-inflammatory cytokines and vigorous activation of neutrophils/macrophages [159,160,161].

The high cost of production represents another major impediment in scaling up the production of AMPs and subsequently putting them into the market. There is also a lack of toxicology studies that influence the development of AMPs, as well as a sparsity of clinical data that often translates to myriad regulatory issues [154]. Therefore, we recognize an evident need for a robust body of evidence before we can truly reorient clinical practice toward AMPs and adequately inform their development and production.

## 5. Conclusions

In the era of surging antimicrobial resistance (which is already a truly global health hazard), AMPs may be a quite parsimonious solution for eliminating resistant and multi-resistant pathogenic microorganisms. Nevertheless, their incorporation into quotidian clinical practice is laden with challenges; thus, only a small number of them are currently used. Some of the reasons behind this are an arduous screening process for effective compounds, bioavailability, potential cytotoxicity issues, costs of production, and regulatory hurdles—but the end goal seems very promising. Such optimism is not unfounded if we take into account the vast pool of potential AMPs, their multitarget and rapid mode of action (frequently showing synergistic interactions with conventional antimicrobial agents), immunomodulatory traits, as well as the lower propensity for antimicrobial resistance development. From the structure of AMPs to the examples of clinical trials and practical uses, we showed how this research field could be exploited to address antimicrobial resistance and solve other pertinent challenges related to human health and well-being.

## Data Availability

Not applicable.

## References

[1] Lei J., Sun L.C., Huang S., Zhu C., Li P., He J., Mackey V., Coy D.H., He Q.Y. (2019). The antimicrobial peptides and their potential clinical applications. Am. J. Transl. Res..

[2] Talapko J., Škrlec I. (2020). The Principles, Mechanisms, and Benefits of Unconventional Agents in the Treatment of Biofilm Infection. Pharmaceuticals.

[3] Burnham J.P. (2021). Climate change and antibiotic resistance: A deadly combination. Ther. Adv. Infect. Dis..

[4] Giacomini E., Perrone V., Alessandrini D., Paoli D., Nappi C., Esposti L.D. (2021). Evidence of Antibiotic Resistance from Population-Based Studies: A Narrative Review. Infect. Drug Resist..

[5] Murray C.J., Ikuta K.S., Sharara F., Swetschinski L., Robles Aguilar G., Gray A., Han C., Bisignano C., Rao P., Wool E. (2022). Global burden of bacterial antimicrobial resistance in 2019: A systematic analysis. Lancet.

[6] Bhattacharjya S., Mohid S.A., Bhunia A. (2022). Atomic-Resolution Structures and Mode of Action of Clinically Relevant Antimicrobial Peptides. Int. J. Mol. Sci..

[7] Shaka M., Arias-Rojas A., Hrdina A., Frahm D., Iatsenko I. (2022). Lipopolysaccharide-mediated resistance to host antimicrobial peptides and hemocyte-derived reactive-oxygen species are the major *Providencia alcalifaciens* virulence factors in *Drosophila melanogaster*. PLoS Pathog..

[8] Bhattacharjya S., Straus S.K. (2020). Design, Engineering and Discovery of Novel α-Helical and β-Boomerang Antimicrobial Peptides against Drug Resistant Bacteria. Int. J. Mol. Sci..

[9] Ghimire J., Guha S., Nelson B.J., Morici L.A., Wimley W.C. (2022). The Remarkable Innate Resistance of *Burkholderia* bacteria to Cationic Antimicrobial Peptides: Insights into the Mechanism of AMP Resistance. J. Membr. Biol..

[10] Gan B.H., Gaynord J., Rowe S.M., Deingruber T., Spring D.R. (2021). The multifaceted nature of antimicrobial peptides: Current synthetic chemistry approaches and future directions. Chem. Soc. Rev..

[11] Marshall J.S., Warrington R., Watson W., Kim H.L. (2018). An introduction to immunology and immunopathology. Allergy Asthma. Clin. Immunol..

[12] Reddy K.V.R., Yedery R.D., Aranha C. (2004). Antimicrobial peptides: Premises and promises. Int. J. Antimicrob. Agents.

[13] Huan Y., Kong Q., Mou H., Yi H. (2020). Antimicrobial Peptides: Classification, Design, Application and Research Progress in Multiple Fields. Front. Microbiol..

[14] León-Buitimea A., Garza-Cárdenas C.R., Garza-Cervantes J.A., Lerma-Escalera J.A., Morones-Ramírez J.R. (2020). The Demand for New Antibiotics: Antimicrobial Peptides, Nanoparticles, and Combinatorial Therapies as Future Strategies in Antibacterial Agent Design. Front. Microbiol..

[15] Larsson D.G.J., Flach C.F. (2022). Antibiotic resistance in the environment. Nat. Rev. Microbiol..

[16] Pollard A.J., Bijker E.M. (2021). A guide to vaccinology: From basic principles to new developments. Nat. Rev. Immunol..

[17] Lachowicz J.I., Szczepski K., Scano A., Casu C., Fais S., Orrù G., Pisano B., Piras M., Jaremko M. (2020). The Best Peptidomimetic Strategies to Undercover Antibacterial Peptides. Int. J. Mol. Sci..

[18] Kapil S., Sharma V. (2021). d-Amino acids in antimicrobial peptides: A potential approach to treat and combat antimicrobial resistance. Can. J. Microbiol..

[19] Moretta A., Scieuzo C., Petrone A.M., Salvia R., Manniello M.D., Franco A., Lucchetti D., Vassallo A., Vogel H., Sgambato A. (2021). Antimicrobial Peptides: A New Hope in Biomedical and Pharmaceutical Fields. Front. Cell. Infect. Microbiol..

[20] Mishra A.K., Choi J., Moon E., Baek K.H. (2018). Tryptophan-Rich and Proline-Rich Antimicrobial Peptides. Molecules.

[21] Sarkar T., Chetia M., Chatterjee S. (2021). Antimicrobial Peptides and Proteins: From Nature’s Reservoir to the Laboratory and Beyond. Front. Chem..

[22] Mahlapuu M., Håkansson J., Ringstad L., Björn C. (2016). Antimicrobial peptides: An emerging category of therapeutic agents. Front. Cell. Infect. Microbiol..

[23] Rima M., Rima M., Fajloun Z., Sabatier J.M., Bechinger B., Naas T. (2021). Antimicrobial Peptides: A Potent Alternative to Antibiotics. Antibiotics.

[24] Zhang Q.Y., Yan Z.B., Meng Y.M., Hong X.Y., Shao G., Ma J.J., Cheng X.R., Liu J., Kang J., Fu C.Y. (2021). Antimicrobial peptides: Mechanism of action, activity and clinical potential. Mil. Med. Res..

[25] Strandberg E., Bentz D., Wadhwani P., Ulrich A.S. (2020). Chiral supramolecular architecture of stable transmembrane pores formed by an α-helical antibiotic peptide in the presence of lyso-lipids. Sci. Rep..

[26] Klubthawee N., Adisakwattana P., Hanpithakpong W., Somsri S., Aunpad R. (2020). A novel, rationally designed, hybrid antimicrobial peptide, inspired by cathelicidin and aurein, exhibits membrane-active mechanisms against *Pseudomonas aeruginosa*. Sci. Rep..

[27] Le C.F., Fang C.M., Sekaran S.D. (2017). Intracellular Targeting Mechanisms by Antimicrobial Peptides. Antimicrob. Agents Chemother..

[28] Sinha S., Zheng L., Mu Y., Ng W.J., Bhattacharjya S. (2017). Structure and Interactions of A Host Defense Antimicrobial Peptide Thanatin in Lipopolysaccharide Micelles Reveal Mechanism of Bacterial Cell Agglutination. Sci. Rep..

[29] Hollmann A., Martinez M., Maturana P., Semorile L.C., Maffia P.C. (2018). Antimicrobial Peptides: Interaction with Model and Biological Membranes and Synergism with Chemical Antibiotics. Front. Chem..

[30] Juhász T., Quemé-Peña M., Kővágó B., Mihály J., Ricci M., Horváti K., Bősze S., Zsila F., Beke-Somfai T. (2021). Interplay between membrane active host defense peptides and heme modulates their assemblies and in vitro activity. Sci. Rep..

[31] Zainal Baharin N.H., Khairil Mokhtar N.F., Mohd Desa M.N., Gopalsamy B., Mohd Zaki N.N., Yuswan M.H., Muthanna A., Dzaraly N.D., Abbasiliasi S., Mohd Hashim A. (2021). The characteristics and roles of antimicrobial peptides as potential treatment for antibiotic-resistant pathogens: A review. PeerJ.

[32] Mink C., Strandberg E., Wadhwani P., Melo M.N., Reichert J., Wacker I., Castanho M.A.R.B., Ulrich A.S. (2021). Overlapping Properties of the Short Membrane-Active Peptide BP100 with (i) Polycationic TAT and (ii) α-helical Magainin Family Peptides. Front. Cell. Infect. Microbiol..

[33] Zhang S., Ma M., Shao Z., Zhang J., Fu L., Li X., Fang W., Gao L. (2021). Structure and Formation Mechanism of Antimicrobial Peptides Temporin B- and L-Induced Tubular Membrane Protrusion. Int. J. Mol. Sci..

[34] Moravej H., Moravej Z., Yazdanparast M., Heiat M., Mirhosseini A., Moosazadeh Moghaddam M., Mirnejad R. (2018). Antimicrobial Peptides: Features, Action, and Their Resistance Mechanisms in Bacteria. Microb. Drug Resist..

[35] Boman H.G. (2003). Antibacterial peptides: Basic facts and emerging concepts. J. Intern. Med..

[36] Fjell C.D., Hiss J.A., Hancock R.E.W., Schneider G. (2011). Designing antimicrobial peptides: Form follows function. Nat. Rev. Drug Discov..

[37] Luo Y., Song Y. (2021). Mechanism of Antimicrobial Peptides: Antimicrobial, Anti-Inflammatory and Antibiofilm Activities. Int. J. Mol. Sci..

[38] Koch A.L. (2003). Bacterial wall as target for attack: Past, present, and future research. Clin. Microbiol. Rev..

[39] Münch D., Sahl H.G. (2015). Structural variations of the cell wall precursor lipid II in Gram-positive bacteria—Impact on binding and efficacy of antimicrobial peptides. Biochim. Biophys. Acta.

[40] Wenzel M., Chiriac A.I., Otto A., Zweytick D., May C., Schumacher C., Gust R., Albada H.B., Penkova M., Krämer U. (2014). Small cationic antimicrobial peptides delocalize peripheral membrane proteins. Proc. Natl. Acad. Sci. USA.

[41] Barreto-Santamaría A., Curtidor H., Arévalo-Pinzón G., Herrera C., Suárez D., Pérez W.H., Patarroyo M.E. (2016). A New Synthetic Peptide Having Two Target of Antibacterial Action in *E. coli* ML35. Front. Microbiol..

[42] Haney E.F., Mansour S.C., Hancock R.E.W. (2017). Antimicrobial Peptides: An Introduction. Methods Mol. Biol..

[43] Di Somma A., Moretta A., Canè C., Cirillo A., Duilio A. (2020). Antimicrobial and Antibiofilm Peptides. Biomolecules.

[44] Yeaman M.R., Yount N.Y. (2003). Mechanisms of antimicrobial peptide action and resistance. Pharmacol. Rev..

[45] Ciumac D., Gong H., Hu X., Lu J.R. (2019). Membrane targeting cationic antimicrobial peptides. J. Colloid Interface Sci..

[46] Milletti F. (2012). Cell-penetrating peptides: Classes, origin, and current landscape. Drug Discov. Today.

[47] Brogden K.A. (2005). Antimicrobial peptides: Pore formers or metabolic inhibitors in bacteria?. Nat. Rev. Microbiol..

[48] Neundorf I. (2019). Antimicrobial and Cell-Penetrating Peptides: How to Understand Two Distinct Functions Despite Similar Physicochemical Properties. Adv. Exp. Med. Biol..

[49] Yan J., Wang K., Dang W., Chen R., Xie J., Zhang B., Song J., Wang R. (2013). Two hits are better than one: Membrane-active and DNA binding-related double-action mechanism of NK-18, a novel antimicrobial peptide derived from mammalian NK-lysin. Antimicrob. Agents Chemother..

[50] Ko S.J., Kang N.H., Kim M.K., Park J., Park E., Park G.H., Kang T.W., Na D.E., Park J.B., Yi Y.E. (2019). Antibacterial and anti-biofilm activity, and mechanism of action of pleurocidin against drug resistant *Staphylococcus aureus*. Microb. Pathog..

[51] Wolz C., Geiger T., Goerke C. (2010). The synthesis and function of the alarmone (p)ppGpp in firmicutes. Int. J. Med. Microbiol..

[52] Otto M. (2006). Bacterial evasion of antimicrobial peptides by biofilm formation. Curr. Top. Microbiol. Immunol..

[53] Maiti B.K. (2020). Potential Role of Peptide-Based Antiviral Therapy against SARS-CoV-2 Infection. ACS Pharmacol. Transl. Sci..

[54] Li X., Zuo S., Wang B., Zhang K., Wang Y. (2022). Antimicrobial Mechanisms and Clinical Application Prospects of Antimicrobial Peptides. Molecules.

[55] Ahmed A., Siman-Tov G., Hall G., Bhalla N., Narayanan A. (2019). Human Antimicrobial Peptides as Therapeutics for Viral Infections. Viruses.

[56] Vilas Boas L.C.P., Campos M.L., Berlanda R.L.A., de Carvalho Neves N., Franco O.L. (2019). Antiviral peptides as promising therapeutic drugs. Cell. Mol. Life Sci..

[57] Skalickova S., Heger Z., Krejcova L., Pekarik V., Bastl K., Janda J., Kostolansky F., Vareckova E., Zitka O., Adam V. (2015). Perspective of Use of Antiviral Peptides against Influenza Virus. Viruses.

[58] Lee H., Lee Y., Kim J., An J., Lee S., Kong H., Song Y., Lee C.K., Kim K. (2018). Modulation of the gut microbiota by metformin improves metabolic profiles in aged obese mice. Gut Microbes.

[59] Barlow P.G., Svoboda P., Mackellar A., Nash A.A., York I.A., Pohl J., Davidson D.J., Donis R.O. (2011). Antiviral activity and increased host defense against influenza infection elicited by the human cathelicidin LL-37. PLoS ONE.

[60] Holani R., Babbar A., Blyth G.A.D., Lopes F., Jijon H., McKay D.M., Hollenberg M.D., Cobo E.R. (2020). Cathelicidin-mediated lipopolysaccharide signaling via intracellular TLR4 in colonic epithelial cells evokes CXCL8 production. Gut Microbes.

[61] Hoffmann J., Schneider C., Heinbockel L., Brandenburg K., Reimer R., Gabriel G. (2014). A new class of synthetic anti-lipopolysaccharide peptides inhibits influenza A virus replication by blocking cellular attachment. Antiviral Res..

[62] Horne W.S., Wiethoff C.M., Cui C., Wilcoxen K.M., Amorin M., Ghadiri M.R., Nemerow G.R. (2005). Antiviral cyclic D,L-alpha-peptides: Targeting a general biochemical pathway in virus infections. Bioorg. Med. Chem..

[63] Mulder K.C.L., Lima L.A., Miranda V.J., Dias S.C., Franco O.L. (2013). Current scenario of peptide-based drugs: The key roles of cationic antitumor and antiviral peptides. Front. Microbiol..

[64] Albiol Matanic V.C., Castilla V. (2004). Antiviral activity of antimicrobial cationic peptides against Junin virus and herpes simplex virus. Int. J. Antimicrob. Agents.

[65] He M., Zhang H., Li Y., Wang G., Tang B., Zhao J., Huang Y., Zheng J. (2018). Cathelicidin-derived antimicrobial peptides inhibit Zika virus through direct inactivation and interferon pathway. Front. Immunol..

[66] Bakovic A., Risner K., Bhalla N., Alem F., Chang T.L., Weston W., Harness J.A., Narayanan A. (2021). Brilacidin Demonstrates Inhibition of SARS-CoV-2 in Cell Culture. Viruses.

[67] Bhattacharya R., Gupta A.M., Mitra S., Mandal S., Biswas S.R. (2021). A natural food preservative peptide nisin can interact with the SARS-CoV-2 spike protein receptor human ACE2. Virology.

[68] Liscano Y., Oñate-Garzón J., Ocampo-Ibáñez I.D. (2020). In Silico Discovery of Antimicrobial Peptides as an Alternative to Control SARS-CoV-2. Molecules.

[69] Zhang R., Jiang X., Qiao J., Wang Z., Tong A., Yang J., Yang S., Yang L. (2021). Antimicrobial peptide DP7 with potential activity against SARS coronavirus infections. Signal Transduct. Target. Ther..

[70] De Cesare G.B., Cristy S.A., Garsin D.A., Lorenz M.C. (2020). Antimicrobial peptides: A new frontier in antifungal therapy. mBio.

[71] Oshiro K.G.N., Rodrigues G., Monges B.E.D., Cardoso M.H., Franco O.L. (2019). Bioactive Peptides against Fungal Biofilms. Front. Microbiol..

[72] Vallabhaneni S., Chiller T.M. (2016). Fungal Infections and New Biologic Therapies. Curr. Rheumatol. Rep..

[73] Chowdhary A., Sharma C., Meis J.F. (2017). Azole-resistant aspergillosis: Epidemiology, molecular mechanisms, and treatment. J. Infect. Dis..

[74] Fisher M.C., Hawkins N.J., Sanglard D., Gurr S.J. (2018). Health and Food Security—TCLocal. Science.

[75] di Luca M., Maccari G., Nifosí R. (2014). Treatment of microbial biofilms in the post-antibiotic era: Prophylactic and therapeutic use of antimicrobial peptides and their design by bioinformatics tools. Pathog. Dis..

[76] Rautenbach M., Troskie A.M., Vosloo J.A. (2016). Antifungal peptides: To be or not to be membrane active. Biochimie.

[77] Fernández de Ullivarri M., Arbulu S., Garcia-Gutierrez E., Cotter P.D. (2020). Antifungal Peptides as Therapeutic Agents. Front. Cell. Infect. Microbiol..

[78] Lucca A.J. (2000). De Expert Opinion on Investigational Drugs Antifungal peptides: Potential candidates for the treatment of fungal infections. Expert Opin. Investig. Drugs.

[79] Bondaryk M., Staniszewska M., Zielińska P., Urbańczyk-Lipkowska Z. (2017). Natural Antimicrobial Peptides as Inspiration for Design of a New Generation Antifungal Compounds. J. Fungi.

[80] Akkam Y. (2016). A review of antifungal peptides: Basis to new era of antifungal drugs. Jordan J. Pharm. Sci..

[81] Cools T.L., Struyfs C., Cammue B.P., Thevissen K. (2017). Antifungal plant defensins: Increased insight in their mode of action as a basis for their use to combat fungal infections. Future Microbiol..

[82] Shafee T.M.A., Lay F.T., Hulett M.D., Anderson M.A. (2016). The Defensins Consist of Two Independent, Convergent Protein Superfamilies. Mol. Biol. Evol..

[83] Parisi K., Shafee T.M.A., Quimbar P., van der Weerden N.L., Bleackley M.R., Anderson M.A. (2019). The evolution, function and mechanisms of action for plant defensins. Semin. Cell Dev. Biol..

[84] Chairatana P., Chiang I.L., Nolan E.M. (2017). Human α-Defensin 6 Self-Assembly Prevents Adhesion and Suppresses Virulence Traits of Candida albicans. Biochemistry.

[85] Ballard E., Yucel R., Melchers W.J.G., Brown A.J.P., Verweij P.E., Warris A. (2020). Antifungal activity of antimicrobial peptides and proteins against *Aspergillus fumigatus*. J. Fungi.

[86] Martinez L.R., Casadevall A. (2006). Cryptococcus neoformans cells in biofilms are less susceptible than planktonic cells to antimicrobial molecules produced by the innate immune system. Infect. Immun..

[87] Zanetti M., Gennaro R., Romeo D. (1995). Cathelicidins: A novel protein family with a common proregion and a variable C-terminal antimicrobial domain. FEBS Lett..

[88] Ridyard K.E., Overhage J. (2021). The potential of human peptide ll-37 as an antimicrobial and anti-biofilm agent. Antibiotics.

[89] Risso A., Braidot E., Sordano M.C., Vianello A., Macrì F., Skerlavaj B., Zanetti M., Gennaro R., Bernardi P. (2002). BMAP-28, an Antibiotic Peptide of Innate Immunity, Induces Cell Death through Opening of the Mitochondrial Permeability Transition Pore. Mol. Cell. Biol..

[90] Scarsini M., Tomasinsig L., Arzese A., D’Este F., Oro D., Skerlavaj B. (2015). Antifungal activity of cathelicidin peptides against planktonic and biofilm cultures of Candida species isolated from vaginal infections. Peptides.

[91] Oppenheim F.G., Xu T., McMillian F.M., Levitz S.M., Diamond R.D., Offner G.D., Troxler R.F. (1988). Histatins, a novel family of histidine-rich proteins in human parotid secretion. Isolation, characterization, primary structure, and fungistatic effects on *Candida albicans*. J. Biol. Chem..

[92] Pusateria C.R., Monacoa E.A., Edgertona M. (2009). Sensitivity of *Candida albicans* Biofilm Cells Grown on Denture Acrylic to Antifungal Proteins and Chlorhexidine. Arch. Oral Biol..

[93] Konopka K., Dorocka-Bobkowska B., Gebremedhin S., Düzgüneş N. (2010). Susceptibility of *Candida* biofilms to histatin 5 and fluconazole. Antonie van Leeuwenhoek Int. J. Gen. Mol. Microbiol..

[94] van der Does A.M., Hiemstra P.S., Mookherjee N. (2019). Antimicrobial Host Defence Peptides: Immunomodulatory Functions and Translational Prospects. Adv. Exp. Med. Biol..

[95] Kang H.K., Lee H.H., Seo C.H., Park Y. (2019). Antimicrobial and Immunomodulatory Properties and Applications of Marine-Derived Proteins and Peptides. Mar. Drugs.

[96] Kumar P., Kizhakkedathu J.N., Straus S.K. (2018). Antimicrobial Peptides: Diversity, Mechanism of Action and Strategies to Improve the Activity and Biocompatibility In Vivo. Biomolecules.

[97] Ehmann D., Koeninger L., Wendler J., Malek N.P., Stange E.F., Wehkamp J., Jensen B.A.H. (2020). Fragmentation of Human Neutrophil α-Defensin 4 to Combat Multidrug Resistant Bacteria. Front. Microbiol..

[98] Guryanova S.V., Ovchinnikova T.V. (2022). Immunomodulatory and Allergenic Properties of Antimicrobial Peptides. Int. J. Mol. Sci..

[99] Ouellette A.J. (2011). Paneth cell α-defensins in enteric innate immunity. Cell. Mol. Life Sci..

[100] Pahar B., Madonna S., Das A., Albanesi C., Girolomoni G. (2020). Immunomodulatory Role of the Antimicrobial LL-37 Peptide in Autoimmune Diseases and Viral Infections. Vaccines.

[101] Meade K.G., O’Farrelly C. (2019). β-Defensins: Farming the Microbiome for Homeostasis and Health. Front. Immunol..

[102] Semple F., Dorin J.R. (2012). β-Defensins: Multifunctional modulators of infection, inflammation and more?. J. Innate Immun..

[103] Ghosh S.K., Feng Z., Fujioka H., Lux R., McCormick T.S., Weinberg A. (2018). Conceptual Perspectives: Bacterial Antimicrobial Peptide Induction as a Novel Strategy for Symbiosis with the Human Host. Front. Microbiol..

[104] Machado L.R., Ottolini B. (2015). An evolutionary history of defensins: A role for copy number variation in maximizing host innate and adaptive immune responses. Front. Immunol..

[105] Candela M.E., Allsop D.J.P., Carter R.N., Semple F., Kilanowski F., Webb S., Taggart D., Mullan H.J., McHugh B.J., Dockrell D.H. (2021). Classical macrophage polarisation is limited by human β-defensin-3 via an autocrine IL-4 dependent process. bioRxiv.

[106] Xu D., Lu W. (2020). Defensins: A Double-Edged Sword in Host Immunity. Front. Immunol..

[107] Zheng D., Liwinski T., Elinav E. (2020). Interaction between microbiota and immunity in health and disease. Cell Res..

[108] Gera S., Kankuri E., Kogermann K. (2021). Antimicrobial peptides—Unleashing their therapeutic potential using nanotechnology. Pharmacol. Ther..

[109] Komatsu T., Watanabe K., Hamada N., Helmerhorst E., Oppenheim F., Lee M.C. (2021). Il Association between Antimicrobial Peptide Histatin 5 Levels and Prevalence of Candida in Saliva of Patients with Down Syndrome. Antibiotics.

[110] Sharma P., Chaudhary M., Khanna G., Rishi P., Kaur I.P. (2021). Envisaging Antifungal Potential of Histatin 5: A Physiological Salivary Peptide. J. Fungi.

[111] Bastos P., Trindade F., da Costa J., Ferreira R., Vitorino R. (2018). Human Antimicrobial Peptides in Bodily Fluids: Current Knowledge and Therapeutic Perspectives in the Postantibiotic Era. Med. Res. Rev..

[112] Norris H.L., Kumar R., Ong C.Y., Xu D., Edgerton M. (2020). Zinc Binding by Histatin 5 Promotes Fungicidal Membrane Disruption in *C. albicans* and *C. glabrata*. J. Fungi.

[113] Lee S.M., Son K.N., Shah D., Ali M., Balasubramaniam A., Shukla D., Aakalu V.K. (2021). Histatin-1 Attenuates LPS-Induced Inflammatory Signaling in RAW264.7 Macrophages. Int. J. Mol. Sci..

[114] van Harten R.M., van Woudenbergh E., van Dijk A., Haagsman H.P. (2018). Cathelicidins: Immunomodulatory Antimicrobials. Vaccines.

[115] Zhang L., Wu W.K.K., Gallo R.L., Fang E.F., Hu W., Ling T.K.W., Shen J., Chan R.L.Y., Lu L., Luo X.M. (2016). Critical Role of Antimicrobial Peptide Cathelicidin for Controlling *Helicobacter pylori* Survival and Infection. J. Immunol..

[116] Benfield A.H., Henriques S.T. (2020). Mode-of-Action of Antimicrobial Peptides: Membrane Disruption vs. Intracellular Mechanisms. Front. Med. Technol..

[117] Lin L., Chi J., Yan Y., Luo R., Feng X., Zheng Y., Xian D., Li X., Quan G., Liu D. (2021). Membrane-disruptive peptides/peptidomimetics-based therapeutics: Promising systems to combat bacteria and cancer in the drug-resistant era. Acta Pharm. Sin. B.

[118] Barańska-Rybak W., Sonesson A., Nowicki R., Schmidtchen A. (2006). Glycosaminoglycans inhibit the antibacterial activity of LL-37 in biological fluids. J. Antimicrob. Chemother..

[119] Alford M.A., Baquir B., Santana F.L., Haney E.F., Hancock R.E.W. (2020). Cathelicidin Host Defense Peptides and Inflammatory Signaling: Striking a Balance. Front. Microbiol..

[120] Choi K.Y.G., Mookherjee N. (2012). Multiple immune-modulatory functions of cathelicidin host defense peptides. Front. Immunol..

[121] Zasloff M. (2002). Antimicrobial peptides of multicellular organisms. Nature.

[122] Dash R., Bhattacharjya S. (2021). Thanatin: An Emerging Host Defense Antimicrobial Peptide with Multiple Modes of Action. Int. J. Mol. Sci..

[123] Upert G., Luther A., Obrecht D., Ermert P. (2021). Emerging peptide antibiotics with therapeutic potential. Med. Drug Discov..

[124] Roberts K.D., Zhu Y., Azad M.A.K., Han M.L., Wang J., Wang L., Yu H.H., Horne A.S., Pinson J.A., Rudd D. (2022). A synthetic lipopeptide targeting top-priority multidrug-resistant Gram-negative pathogens. Nat. Commun..

[125] Thakur A., Sharma A., Alajangi H.K., Jaiswal P.K., Lim Y.-B., Singh G., Barnwal R.P. (2022). In pursuit of next-generation therapeutics: Antimicrobial peptides against superbugs, their sources, mechanism of action, nanotechnology-based delivery, and clinical applications. Int. J. Biol. Macromol..

[126] Mehta K., Sharma P., Mujawar S., Vyas A. (2022). Role of Antimicrobial Peptides in Treatment and Prevention of Mycobacterium Tuberculosis: A Review. Int. J. Pept. Res. Ther..

[127] Stevenson C. (2009). Advances in peptide pharmaceuticals. Curr. Pharm. Biotechnol..

[128] Zavascki A.P., Goldani L.Z., Li J., Nation R.L. (2007). Polymyxin B for the treatment of multidrug-resistant pathogens: A critical review. J. Antimicrob. Chemother..

[129] Sierra J.M., Fusté E., Rabanal F., Vinuesa T., Viñas M. (2017). An overview of antimicrobial peptides and the latest advances in their development. Expert Opin. Biol. Ther..

[130] Dijksteel G.S., Ulrich M.M.W., Middelkoop E., Boekema B.K.H.L. (2021). Review: Lessons Learned From Clinical Trials Using Antimicrobial Peptides (AMPs). Front. Microbiol..

[131] Sakamoto I., Igarashi M., Kimura K., Takagi A., Miwa T., Koga Y. (2001). Suppressive effect of *Lactobacillus gasseri* OLL 2716 (LG21) on *Helicobacter pylori* infection in humans. J. Antimicrob. Chemother..

[132] Mitra D., Yadav A., Prithyani S., John L.E., Rodrigues S., Shah R. (2019). The antiplaque efficacy of lantibiotic Nisin extract mouthrinse. J. Indian Soc. Periodontol..

[133] Assmann T.S., Cuevas-Sierra A., Salas-Pérez F., Riezu-Boj J.I., Milagro F.I., Martínez J.A. (2020). Crosstalk between circulating microRNAs and chronotypical features in subjects with metabolic syndrome. Chronobiol. Int..

[134] Håkansson J., Ringstad L., Umerska A., Johansson J., Andersson T., Boge L., Rozenbaum R.T., Sharma P.K., Tollbäck P., Björn C. (2019). Characterization of the in vitro, ex vivo, and in vivo Efficacy of the Antimicrobial Peptide DPK-060 Used for Topical Treatment. Front. Cell. Infect. Microbiol..

[135] van der Velden W.J.F.M., van Iersel T.M.P., Blijlevens N.M.A., Donnelly J.P. (2009). Safety and tolerability of the antimicrobial peptide human lactoferrin 1-11 (hLF1-11). BMC Med..

[136] Martin-Loeches I., Dawgul M. (2017). Antimicrobial Peptides under Clinical Trials. Curr. Top. Med. Chem..

[137] Martin-Loeches I., Dale G.E., Torres A. (2018). Murepavadin: A new antibiotic class in the pipeline. Expert Rev. Anti. Infect. Ther..

[138] Wiig M.E., Dahlin L.B., Fridén J., Hagberg L., Larsen S.E., Wiklund K., Mahlapuu M. (2014). PXL01 in sodium hyaluronate for improvement of hand recovery after flexor tendon repair surgery: Randomized controlled trial. PLoS ONE.

[139] Grönberg A., Mahlapuu M., Ståhle M., Whately-Smith C., Rollman O. (2014). Treatment with LL-37 is safe and effective in enhancing healing of hard-to-heal venous leg ulcers: A randomized, placebo-controlled clinical trial. Wound Repair Regen..

[140] Mahlapuu M., Sidorowicz A., Mikosinski J., Krzyżanowski M., Orleanski J., Twardowska-Saucha K., Nykaza A., Dyaczynski M., Belz-Lagoda B., Dziwiszek G. (2021). Evaluation of LL-37 in healing of hard-to-heal venous leg ulcers: A multicentric prospective randomized placebo-controlled clinical trial. Wound Repair Regen..

[141] Hancock R.E.W., Haney E.F., Gill E.E. (2016). The immunology of host defence peptides: Beyond antimicrobial activity. Nat. Rev. Immunol..

[142] Lu F., Zhu Y., Zhang G., Liu Z. (2022). Renovation as innovation: Repurposing human antibacterial peptide LL-37 for cancer therapy. Front. Pharmacol..

[143] Tokajuk J., Deptuła P., Piktel E., Daniluk T., Chmielewska S., Wollny T., Wolak P., Fiedoruk K., Bucki R. (2022). Cathelicidin LL-37 in Health and Diseases of the Oral Cavity. Biomedicines.

[144] Baker J.L., He X., Shi W. (2019). Precision Reengineering of the Oral Microbiome for Caries Management. Adv. Dent. Res..

[145] Elad S., Epstein J.B., Raber-Durlacher J., Donnelly P., Strahilevitz J. (2012). The antimicrobial effect of Iseganan HCl oral solution in patients receiving stomatotoxic chemotherapy: Analysis from a multicenter, double-blind, placebo-controlled, randomized, phase III clinical trial. J. Oral Pathol. Med..

[146] Blakaj A., Bonomi M., Gamez M.E., Blakaj D.M. (2019). Oral mucositis in head and neck cancer: Evidence-based management and review of clinical trial data. Oral Oncol..

[147] Zhao C., Yan S., Song Y., Xia X. (2022). Roles of Antimicrobial Peptides in Gynecological Cancers. Int. J. Mol. Sci..

[148] Gombart A.F., Borregaard N., Koeffler H.P. (2005). Human cathelicidin antimicrobial peptide (CAMP) gene is a direct target of the vitamin D receptor and is strongly up-regulated in myeloid cells by 1,25-dihydroxyvitamin D3. FASEB J..

[149] Liu P.T., Stenger S., Tang D.H., Modlin R.L. (2007). Cutting edge: Vitamin D-mediated human antimicrobial activity against *Mycobacterium tuberculosis* is dependent on the induction of cathelicidin. J. Immunol..

[150] Briceno Noriega D., Savelkoul H.F.J. (2022). Vitamin D: A Potential Mitigation Tool for the Endemic Stage of the COVID-19 Pandemic?. Front. Public Health.

[151] Dixon B.M., Barker T., McKinnon T., Cuomo J., Frei B., Borregaard N., Gombart A.F. (2012). Positive correlation between circulating cathelicidin antimicrobial peptide (hCAP18/LL-37) and 25-hydroxyvitamin D levels in healthy adults. BMC Res. Notes.

[152] Han J.E., Alvarez J.A., Jones J.L., Tangpricha V., Brown M.A., Hao L., Brown L.A.S., Martin G.S., Ziegler T.R. (2017). Impact of high-dose vitamin D 3 on plasma free 25-hydroxyvitamin D concentrations and antimicrobial peptides in critically ill mechanically ventilated adults. Nutrition.

[153] Sidorczuk K., Gagat P., Pietluch F., Kała J., Rafacz D., Bąkała L., Słowik J., Kolenda R., Rödiger S., Fingerhut L.C.H.W. (2022). Benchmarks in antimicrobial peptide prediction are biased due to the selection of negative data. Brief. Bioinform..

[154] Vlieghe P., Lisowski V., Martinez J., Khrestchatisky M. (2010). Synthetic therapeutic peptides: Science and market. Drug Discov. Today.

[155] Makowski M., Silva Í.C., Do Amaral C.P., Gonçalves S., Santos N.C. (2019). Advances in lipid and metal nanoparticles for antimicrobial peptide delivery. Pharmaceutics.

[156] Zharkova M.S., Orlov D.S., Golubeva O.Y., Chakchir O.B., Eliseev I.E., Grinchuk T.M., Shamova O.V. (2019). Application of antimicrobial peptides of the innate immune system in combination with conventional antibiotics—A novel way to combat antibiotic resistance?. Front. Cell. Infect. Microbiol..

[157] Greber K.E., Roch M., Rosato M.A., Martinez M.P., Rosato A.E. (2020). Efficacy of newly generated short antimicrobial cationic lipopeptides against methicillin-resistant *Staphylococcus aureus* (MRSA). Int. J. Antimicrob. Agents.

[158] Lesiuk M., Paduszyńska M., Greber K.E. (2022). Synthetic Antimicrobial Immunomodulatory Peptides: Ongoing Studies and Clinical Trials. Antibiotics.

[159] Haney E.F., Hancock R.E.W. (2013). Peptide design for antimicrobial and immunomodulatory applications. Biopolymers.

[160] Hilchie A.L., Wuerth K., Hancock R.E.W. (2013). Immune modulation by multifaceted cationic host defense (antimicrobial) peptides. Nat. Chem. Biol..

[161] Drayton M., Deisinger J.P., Ludwig K.C., Raheem N., Müller A., Schneider T., Straus S.K. (2021). Host Defense Peptides: Dual Antimicrobial and Immunomodulatory Action. Int. J. Mol. Sci..

